# HIV and Older Adults: Evolving Into a Manageable Condition

**DOI:** 10.1093/geroni/igaa057.3112

**Published:** 2020-12-16

**Authors:** Charles Emlet

**Affiliations:** University of Washington, Tacoma, Tacoma, Washington, United States

## Abstract

I was fortunate to serve at the HIV and Aging Interest Convener from 2000 until 2008 and co-convener from 2009 until 2012. During this period, we began to see the full and positive impact of Highly Active Antiretroviral Treatments creating highly sought longevity for older adults. During that time, the numbers of people over the age of 50, living with HIV, climbed from an estimated 81,103 to 330,843. HIV was identified by President Clinton as a national security threat while the CDC unveiled a new plan for HIV prevention. In 2006 the CDC released revised HIV testing recommendations for those 18-64 years of age to include opt out testing, stopping short of recommending HIV testing for older people. HIV sessions at GSA’s Annual Scientific meeting continued to broaden and evolve. Part of a symposium sponsored by the HIV, AIDS and Older Adults Interest Group.

